# Experimental Outcomes of the Mediterranean Diet: Lessons Learned from the Predimed Randomized Controlled Trial

**DOI:** 10.3390/nu11122991

**Published:** 2019-12-06

**Authors:** Dicle Kargin, Laura Tomaino, Lluís Serra-Majem

**Affiliations:** 1 Research Institute of Biomedical and Health Sciences, University of Las Palmas de Gran Canaria, 35016 Las Palmas de Gran Canaria, Spain; dicle.kargin@marmara.edu.tr (D.K.); laura.tomaino@unimi.it (L.T.); 2 Department of Nutrition and Dietetics, Institute of Health Sciences, Marmara University, 34854 Istanbul, Turkey; 3 Department of Clinical and Community Health (DISCCO), Universita’ degli Studi di Milano, 20122 Milan, Italy; 4 Complejo Hospitalario Universitario Insular-Materno Infantil (CHUIMI), Canarian Health Services, 35016 Las Palmas de Gran Canaria, Spain; 5 Consorcio CIBER, M.P. Fisiopatologia de la Obesidad y Nutricion (CIBERObn), Instituto de Salud Carlos III (ISCIII), 28029 Madrid, Spain

**Keywords:** PREDIMED, Mediterranean diet, dietary intervention, randomized controlled trials, cardiovascular disease, type-2 diabetes mellitus, metabolic syndrome

## Abstract

The Mediterranean Diet (MD) is, culturally and historically, the nutritional pattern shared by people living in the olive-tree growing areas of the Mediterranean basin. It is of great importance for its potential preventive effect against cardiovascular diseases (CVDs). The PREvención con DIeta MEDiterránea (PREDIMED) study, a Spanish multicentre randomised controlled trial (RCT), was designed to assess the long-term effects of the MD, without any energy restriction, on the incidence of CVD in individuals at high cardiovascular (CV) risk. Since its inception, it gave a great contribution to the available literature on the issue. It is well known that, in the field of the health sciences, RCTs provide the best scientific evidence. Thus, the aim of the present review is to analyse the results of the RCTs performed within the frame of the PREDIMED study. Our findings showed that MD has beneficial effects in the primary prevention of CVDs, diabetes and in the management of metabolic syndrome.

## 1. Introduction

The Mediterranean diet (MD) is a nutritional model proposed by Ancel Keys, based on the dietary traditions shared around the fifties (1950s) by populations that inhabited the Hellenic peninsula, Italy, and the other countries overlooking the Mediterranean Sea [1]. In descriptive terms, MD is the dietary pattern historically and culturally prevailing among people residing in the olive tree-growing areas of the Mediterranean region before globalization made its effect on lifestyle, diet included [1,2]. Even if the different regions in these areas have their own dietary traditions, they could be considered as variants of the most comprehensive MD [3]. Graphically, it is represented by a pyramid that represents food according to their frequency of intake: rarely to often (weekly or daily), from the basis to the apex, respectively [4].

The MD model is closely related to the history of civilization of the areas surrounding the Mediterranean Sea, and the foods characterizing this dietary pattern have been part of the diet and consumed since many centuries ago. In ancient times, the staple food of the populations residing in the setting of the Mediterranean Sea were non-starchy vegetables (present in abundancy and assortment), minimally processed whole-grain cereals, legumes, nuts, and seeds [5]. Nowadays, the MD is composed by plentiful use of olive oil, high consumption of fruit, vegetables, legumes, cereals and nuts, regular but moderate intake of wine (especially red wine) with meals, moderate consumption of fish, seafood, fermented dairy products (yogurt and cheese), poultry and eggs; and limited consumption of red and processed meats and sweets [6].

However, the investigation of the MD’s effects on health did not begin until the 20th century. The first study to observe a protective effect of the MD or some of its components was the Seven Countries Study [7]. It reported a strong inverse association between monounsaturated fatty acid intake (the main source of fat from olive oil, essential component of the MD) and overall mortality, especially due to coronary heart disease (CHD) and cancer. Afterwards, MD and its effects on health were mostly investigated by means of observational studies and personal reviews, with the exception of the Lyon Heart Study in France, which revealed that modified MDs were associated with remarkable reductions in CHD event rates and cardiovascular (CV) mortality [8], and other small scale clinical trials [9]. In recent times, the number of randomized controlled trials (RCTs) and meta-analyses increased significantly, with the objective to examine the impact of the MD on various health outcomes [10].

The MD pattern reached considerable importance due to its role in the prevention of cardiovascular diseases (CVDs). The inverse association between adherence to MD and CVD mortality, reported by Seven Countries study [7], paved the way for the increasing importance that MD acquired in cardiovascular epidemiology [1,7]. As a result, the American Heart Association qualified the Mediterranean Food Pattern as potentially effective for the prevention of CHD, though emphasizing the need of more studies before suggesting people to pursue a MD pattern [11]. 

Although the first references to the benefits of MD on health focused on the protective effect against CVDs, its effects on other health issues were later investigated. For instance, the available literature reports the inverse association between specific nutrients, food components and the Mediterranean dietary pattern, and several health conditions, such as: Specific types of cancer, diabetes mellitus, obesity, cognitive decline and mental health, respiratory diseases, osteoarthritis, and quality of life or healthy aging [10].

To date, several studies have been conducted in Spain and other Mediterranean countries in the scope of MD and its relationship with health, and the evidence of the beneficial role of this pattern is being constantly enhanced [12]. The PREvención con DIeta MEDiterránea (PREDIMED) study is a primary prevention multicentre randomised controlled trial (RCT) designed to test the hypothesis that the MD would be superior to a low-fat diet for CVD protection in asymptomatic patients at high CV risk [13].

### The PREDIMED Study

The PREDIMED study is a large, parallel group, multicentre, randomized, controlled, nutritional intervention trial designed to assess the effects of the Mediterranean Diet on the primary prevention of CVD (www.predimed.es) [14]. The study was conducted in Spain from 2003 to 2011 and was funded exclusively by Instituto de Salud Carlos III, while food industries provided Extra Virgin Olive Oil (EVOO) and nuts free of charge. 

The protocol, design and methods of the trial have been reported previously [15,16] and their detailed description goes beyond our objectives. To sum up, community-dwelling men (aged 55–80 years old) and women (aged 60–80) without predetermined diagnosis of CVD were included in the study, and were considered acceptable to participate if they had either type 2 diabetes mellitus (DM) or ≥ 3 of the following major CV risk factors: hypertension, high plasma low-density lipoprotein (LDL) cholesterol, low plasma high-density lipoprotein (HDL) cholesterol, overweight or obesity (BMI ≥ 25 kg/m^2^), current history of smoking and family history of premature CHD. The enlistment period lasted from October 2003 to June 2009, and enrolled 7447 participants that were randomly assigned to one of the three nutritional intervention groups (ratio 1:1:1) in the Spanish Primary Care Centres affiliated to 11 recruiting centres. Two groups were prescribed a MD enriched with either Extra Virgin Olive Oil (EVOO) (*n* = 2543) or nuts (walnuts, almonds and hazelnuts) (*n* = 2454), and the third group (control) was prescribed a low-fat diet (*n* = 2450). None of the three dietary protocols included in the trial provided energy restrictions, and no intervention on participants’ physical activity status was performed. 

Validated food frequency questionnaires covering 137 food items plus vitamin/minerals supplements were collected yearly by trained dietitians, and adherence to the MD was assessed through a 14-items questionnaire [17]. Fasting blood and urine samples were obtained, and serum, plasma and DNA specimens were stored. Biomarkers of adherence to the supplemental foods (urinary hydroxytirosol as marker of EVOO consumption and plasma α-linolenic acid as marker of walnut consumption) were determined in random sub-samples [18].

In addition to the institutional review board of the Hospital Clinic in Barcelona, Spain (approved on 16 July 2002), the institutional review boards of each recruitment centre also approved the study protocol, and participants gave their written informed consent.

The primary aim of the trial was to assess the effects of two MDs (MD + EVOO or MD + nuts) on a composite endpoint of cardiovascular death, myocardial infarction and stroke (primary outcome), compared to a low-fat control diet. Secondary endpoints were: death of any cause, incidence of heart failure, DM, dementia or other neurodegenerative disorders, and major cancers (colorectal, breast, lung, stomach and prostate). To better assess the impact of dietary changes on the risk of clinical events, intermediate outcomes were also evaluated, for instance changes in blood pressure (BP), blood lipids levels, fasting glycaemia, weight gain, and markers of inflammation [16].

According to the pyramid of evidence relative to health science, randomized clinical trials (RCTs) provide the best, and most robust and accountable scientific evidence [19]. Thus, the aim of the present paper is to review and analyse the results of the main and secondary outcomes, as well as the *post hoc* analyses within the frame of the PREDIMED study. 

## 2. Materials and Methods 

The research was conducted in PubMed, and included studies published from February 2006 to August 2019. The MeSH term “PREDIMED” was used as a key word. Titles and abstracts were independently scanned to include all potential studies identified as a result of the researches. The exclusion criteria were: studies not carried out within the scope of PREDIMED, protocols, letters, commentaries, reviews, studies related to PREDIMED-Plus and studies written in languages other than English. We obtained information for the following variables: number of participants at baseline and at the end of the intervention, characteristics of the participants, duration of the intervention, main objective of the intervention, and conclusions, as they appeared in the article.

## 3. Results

**Figure 1:**
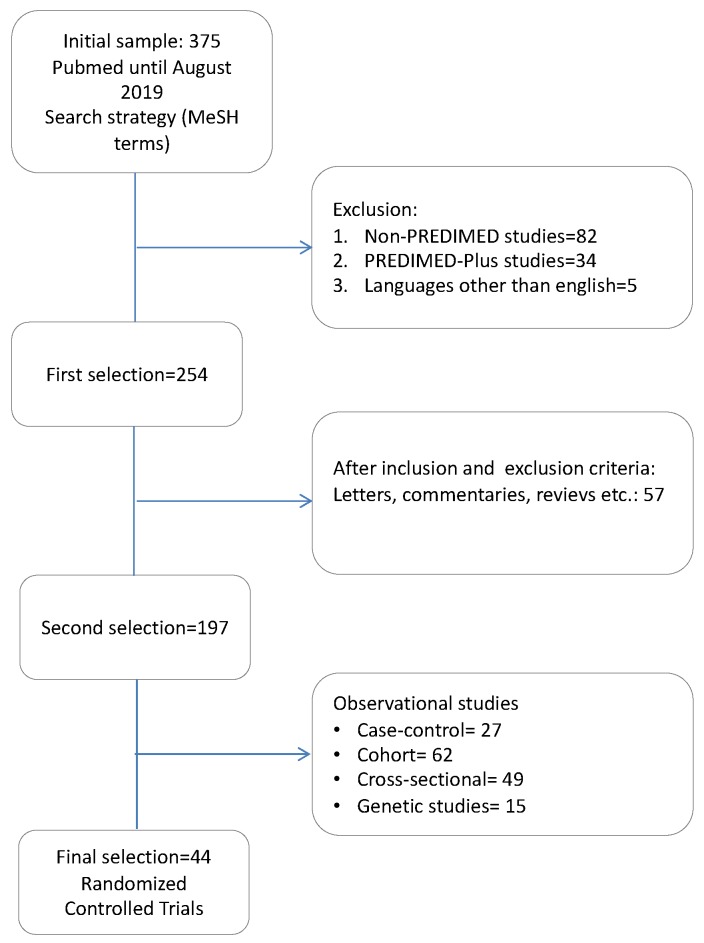
Flow chart of the studies’ selection process.

The PubMed search resulted in 375 abstracts. After applying the exclusion criteria, 197 articles remained for analysis. Since the main purpose of our review was to examine only experimental studies, we excluded observational studies, including cross-sectional, case control and cohort studies, as shown in Figure 1. 

The main characteristics of the 44 randomized controlled PREDIMED studies and their effects on CVDs and other health outcomes included in our review are shown in Table 1, Table 2 and Table 3.

In the PREDIMED study, a total of 8713 candidates were screened for eligibility, and 7447 of them were enrolled and assigned to one of the three intervention groups (MD + EVOO, MD + Nuts or low-fat diet). Their baseline characteristics according to intervention group are described elsewhere [15]. The exclusion of participants whose randomization procedures were known to have deviated from the protocol did not materially change these results [15]. Participants were followed for a median of 4.8 years (interquartile range: 2.8–5.8). When compliance with diet intervention was examined, an increase in the 14-item MD questionnaire score was observed for the two MD groups during the follow-up period. Substantial differences between the MD groups and the control group in 12 of the 14 items of the questionnaire were observed. Also, biomarkers’ level variations indicated good adherence to the dietary assignments [15]. The main nutrient changes in the MD groups reflected the fat content and composition of the supplementary foods (EVOO or nuts). No relevant diet-related adverse effects were reported. Besides, a little difference in physical activity (assessed with specific questionnaires) among the three groups was observed [15].

As the main objective of the PREDIMED study was to examine the effects of MD on the primary prevention of CVDs, the majority of the RCTs included in our review dealt with CVDs and the related risk factors (Table 1). Estruch et al.’s intention to treat analysis, which included all the 7447 participants, revealed a relative risk reduction of 31% for the MD + EVOO (HR 0.69, 95%CI 0.53, 0.91), and 28% MD + Nuts group (HR 0.72, 95%CI 0.54, 0.95) in the primary composite outcome investigated (including acute myocardial infarction, stroke, or death for CV events), compared to the low-fat control diet group [15]. Moreover, Martínez-González et al., observed that the Hazard Ratio, HR (95% Confidence Interval, CI) for atrial fibrillation in the MD + EVOO group was 0.62 (0.45, 0.85), *p* < 0.05 [22]. 

When the effect of MD on diabetes was examined, it was observed that the HR (95% CI) of diabetes incidence was was 0.60 (0.43, 0.85) for the subjects following MD + EVOO compared to controls, and 0.82 (0.61, 1.10) for the MD + Nuts group compared to control diet [36]. After the application of the Fine and Gray model for competing risk analysis, the results remained essentially unchanged [61]. Similarly, a subgroup analysis on the PREDIMED population (*n* = 418), showed a protective effect of the MD either supplemented with EVOO or nuts against the incidence of DM (HR, 95%CI for both MDs versus control 0.47 (0.26–0.87) [62,63]. Another study showed a significant effect of MD on the incidence of diabetic retinopathy: HR (95% CI) 0.59 (0.37, 0.95) for the MD groups [35]. 

Further trials evaluated the long-term effect of MD on incidence and reversion of MetS. Although there were no significant differences in incidence or reversion HRs by intervention, reversion occurred in 958 (28.2%) participants when considering only those subjects who had MetS at baseline [38]. Salas-Salvadó et al., examined the one-year effect of the MD on metabolic syndrome (MetS) status, as shown in Table 2. They found that, after 1-year follow-up, the MetS prevalence was reduced by a 6.7%, 13.7% and 2% in the MD + EVOO, MD + Nuts and control groups, respectively (MD + Nuts versus control group, *p* < 0.05). These differences may be due to the variations in incidence rates among subjects without MetS at baseline and in reversion rates among those who had the syndrome at the beginning of the trial [39]. 

Álvarez-Pérez et al., [41] found that MD had positive effects on body composition and anthropometric measurements in a subsample of the cohort. Nevertheless, no between-group statistically significant differences were found in anthropometric or body composition variables. 

After analysing the influence of a Mediterranean dietary pattern on plasma total antioxidant capacity (TAC), the MD + EVOO group showed higher levels of plasma TAC and a reduction in body weight gain [42].

The effects of the MD on cognitive functions were also examined, as shown in Table 3. In a sub-study conducted on 522 participants in Navarra, it was found that the MD improved cognitive function, assessed with the Mini-Mental State Examination and the clock drawing test [43]. Likewise, another study observed that a long-term intervention with an EVOO-rich MD resulted in a better cognitive function in comparison with controls [44].

Toledo et al.’s study, aimed at investigating the incidence of breast cancer on the PREDIMED population, showed a HR (95% CI) of 0.38 (0.16, 0.87) for the MD + EVOO compared to the control group [53]. Other studies examining the effects of the MD on different conditions, other than CVDs, diabetes obesity and cognitive function, are reported in Table 3.

**Figure 2:**
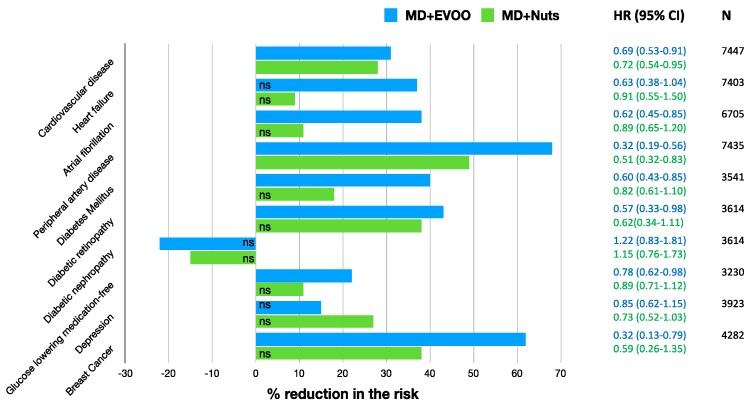
Percentage reduction in the risk of different medical conditions in the PREDIMED Study, according to the group of treatment (MD + EVOO or MD + Nuts) versus the low-fat control diet. The % of risk reduction were computed as: 100 × (1–HR)% and it represents the reduction in the instantaneous risk of the above mentioned events at any given point of time, or the reduction in the rate of such events. ns: not significant. MD: Mediterranean Diet; EVOO: Extra Virgin Olive Oil.

In order to outline the results obtained by the trials analysed in the present review, we calculated the percentage reduction of the risk of various clinical conditions, as shown in Figure 2.

The % reduction in the risk of cardiovascular disease (a composite of death for cardiovascular cause, non-fatal acute myocardial infarction, and non-fatal stroke) was 31% (95% CI 47–9%) and 28% (95% CI 46–5%) for MD + EVOO and MD + Nuts groups, respectively [15]. Nevertheless, it is appropriate to observe that, although the % risk of CVD reduction vary according to the dietary intervention, it is not possible to infer that one is better than the other, as shown by the overlapping of the correspondent 95% confidence intervals. For the heart failure (HF), the % reduction observed was not significant in the MD + EVOO nor in the MD + Nuts [21], that is to say, none of the two dietary interventions turned out to be better than the control diet in the risk reduction of the outcome. For the atrial fibrillation the % risk reduction was 38% (95% CI 55–15%) for the MD + EVOO group, while not significant for the MD + Nuts group [22]. The risk reduction of peripheral artery disease was 68% (95% CI 81–44%) and 49% (95% CI 68–17%) for the MD + EVOO and MD + Nuts groups, respectively [29], but the difference between the two dietary interventions was not statistically significant due to the partial overlapping of the 95% CIs. For the probability of remaining free of the glucose-lowering medications, a reduction of 22% (95% CI 38–2%) was observed for the MD + EVOO; no significance was observed for the MD + Nuts group [33]. The reduction in the risk of diabetic retinopathy was significant only for the MD + EVOO group (43%, 95% CI: 67–2%) but not for the MD + Nuts group [35]. Interestingly, the long-term effect of MD on diabetic nephropathy was not beneficial, probably due to the higher salt intake than a hyposodic diet (Table 2) [35]. For the incidence of diabetes mellitus, the risk reduction was 40% (95% CI 57%, 15%) and 18% (39%, −10%) for the MD + EVOO and MD + Nuts intervention groups respectively [36], and the difference between the two dietary approaches did not turn out to be statistically significant. For the depression risk, the MD supplemented with either EVOO or Nuts did not lead to a significant reduction, compared to the control diet. However, a risk reduction was observed in the Nuts + MD group among the diabetic subjects only [45]. Finally, the % reduction in the risk of breast cancer incidence was 68% (87–21%) for the MD + EVOO group versus the low-fat control diet, while the MD + Nuts did not show to be statistically significant compared to the control group [53]. 

Overall, the MD + EVOO dietary intervention seemed to have more beneficial effects in terms of % reduction of the risk of different clinical condition. However, in those conditions where both MD + EVOO and MD + Nuts had significative effects compared to the control diet, it is not possible to conclude that the former is better than the latter.

Table 4 shows the percentage reduction from baseline of different continuous variables assessed by the different randomized controlled trials conducted in the scope of the PREDIMED study. 

## 4. Discussion

The RCTs conducted within the frame of the PREDIMED study are the study designs able to best describe the effects of the MD on CVDs and other secondary health outcomes, in terms of sample size, duration of the intervention and follow-up. Nevertheless, in a comprehensive review evaluating the epidemiological and molecular aspects of the MD for non-PREDIMED articles, it was emphasized that only few of them evaluated hard endpoints, and that most of the studies had a sample size smaller than 200 people [1]. It was specified that the most convenient study in terms of number of participants, duration of the intervention and number of publications produced was the PREDIMED study [1]. In the present review, 44 RCTs of PREDIMED study met our inclusion criteria, and the majority of them presented a sample size larger than 200 subjects. The aim of the present review is to summarize the results of RCTs in the PREDIMED study, mainly related to cardiovascular diseases, diabetes, obesity, metabolic syndrome and many other important conditions, and to synthetize the best evidence available.

The results of the PREDIMED study reported in 2013 have been partially retracted due to protocol deviations, mainly regarding the randomization process. Nevertheless, after re-analyzing the collected data with the appropriate corrections (omitting 1588 participants whose study group assignment was known or suspected to have deviated from the protocol), the results obtained were similar [15]. 

When both the MD groups (MD + EVOO and MD + Nuts) were examined, the MD nutrition model used in the PREDIMED study turned out to potentially reduce the number of hard clinical events in a relatively short time [18]. Firstly, in 2013 it was reported that both intervention groups showed approximately a 30% reduction in the rate of major CV events (myocardial infarction, stroke or death for CV causes), compared to the control group, after a median follow-up of 4.8 years [13].

The epidemiological evidence of the CVD protection provided by the adherence to the MD is strong. A meta-analysis by Liyanage et al., found that the MD was associated with a 37% relative reduction (*p* < 0.001) in the risk of major CV events [64]. These findings are in agreement with the results of the trials included in the present review, which showed positive effects of the MD on atrial fibrillation [22], and peripheral artery disease [29]. The underlying mechanisms of protection against CVD provided by the MD can be attributed to the abundance of antioxidant and anti-inflammatory molecules in its individual components such as fruits and vegetables, olive oil, nuts, whole grains, fish and red wine, although the specific protective mechanisms of MD on CVDs are not completely understood. One of the hypotheses suggests a possible role of the cell redox state in the modulation of the enzymatic systems related to the antioxidant capacity. Additionally, nutrients have the ability to regulate gene expression and protein synthesis. As reported by nutrigenomic studies, MD can play a role against the expression of several proatherogenic genes involved in vascular inflammation, foam cell formation and thrombosis [18]. 

As secondary endpoints of the PREDIMED study, diabetes incidence and MetS status were also assessed. The largest trial on the incidence of type 2 diabetes mellitus (T2DM) in the primary prevention PREDIMED study, reported a significant reduction of the incidence in both the intervention groups [36]. Moreover, the results of prospective cohort studies contributing to estimate T2DM risk according to different levels of MD adherence provided additional and consistent evidence [65]. Their results support the protective role of the MD against T2DM, with overall risk reductions ranging from 12% to 83% for subjects closely adhering to the MD compared to those reporting the lowest adherence, after adjusting for several confounders [65]. The authors also observed that higher adherence to the MD had a beneficial role in the prevention and treatment of MetS and its components [65]. In the PREDIMED study, although no differences in the onset of MetS were observed among the three groups, participants in the MD + EVOO and MD + Nuts were more likely to present disease reversion, if compared to the control group [38]. Esposito et al., (2015) specified that two meta-analyses assessed the relationship between adherence to a MD and future incidence of diabetes. According to their report, the analyses are consistent with a significant reduction, ranging from 19% to 23%, of new diabetes diagnosis associated with greater adherence to the MD [66]. In the Framingham Heart Study Offspring Cohort, 1918 participants free of the condition at baseline were followed for seven years, and participants in the highest quintile category of the Mediterranean-style dietary pattern score had a lower incidence of metabolic syndrome than those in the lowest quintile category (*p* = 0.01) [67]. It is thought that highly important bioactive components of the MD such as unsaturated fatty acids, complex carbohydrates and fibre, vegetable protein, non-sodium minerals, phytosterols and polyphenols interact synergistically to advantageously affect various metabolic pathways at risk of MetS, T2DM and CVD [65].

The role of MD in the protection against cognitive decline, is being supported by growing evidence. Although the majority of the available studies in the issue present a longitudinal or a cross-sectional design, they point out the protective role of MD on cognitive impairment, cognitive function and decline [68].

Among the secondary outcomes of the PREDIMED study, the incidence of breast cancer was assessed. To date, the evidence on the role of Mediterranean diet in the onset of this neoplasm is still limited; nevertheless, the findings of Toledo et al.’s study (2015) are in agreement with the available literature [69,70], and are statistically strengthened by its prospective, randomized and controlled design. 

As a result, with the exception of the PREDIMED study, most of the studies on MD appear to be observational studies or short-term trials. Among many issues, the findings of the PREDIMED study include a large number of randomized controlled trials that provide a higher level of scientific evidence than cohort studies and represent the gold standard to clarify the actual effects of this intervention. The PREDIMED trial is a milestone of nutrition intervention that indicated with powerful evidence the benefits of the traditional MD in the primary prevention of CVD in individuals at high risk. As secondary endpoints of the PREDIMED study, it was observed that MD interventions could protect against diabetes in participants without diabetes and figure out a role in preventing or managing MetS. and certain metabolic abnormalities that predicts diabetes and cardiometabolic risk. 

## 5. Conclusions

In conclusion, the contribution of the PREDIMED study as a commendable dietary intervention study is certain. This trial present as primary endpoint a composite of CV events and, in the frame of the study, sub-group analyses have been performed to assess various secondary outcomes. The scope of this review was to sum up the experimental outcomes of those studies. Randomized controlled trials within the scope of the PREDIMED study demonstrated the risk-reducing effects on major health problems and risk factors as well as the current and known effects of the Mediterranean diet. When the diet is considered as the main determinant of many health outcomes, we testify the Mediterranean diet as a comprehensive diet model that overcomes a single food or single nutrient approach. 

## Figures and Tables

**Table 1:** Characteristics of the RCTs conducted within the frame of the PREDIMED study, investigating the role of Mediterranean Diet (MD) on cardiovascular disease (CVD) and cardiovascular risk factors.

Aim of the Study	Number of Subjects	Follow-Up Median (Years)	Main Results of the Study	1st Author, Journal, Year	Ref.
**Cardiovascular Disease**					
Incidence of primary endpoint (a composite of CV events: Non-fatal acute myocardial infarction, non-fatal stroke or death from CV causes)	7447	4.8	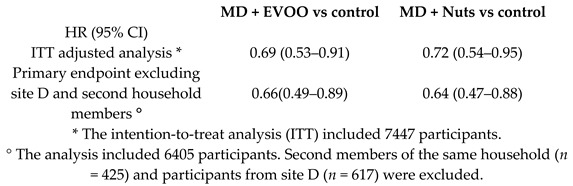	Estruch et al. *N. Engl. J. Med.* 2018	[15]
Incidence of heart failure	7403	4.8	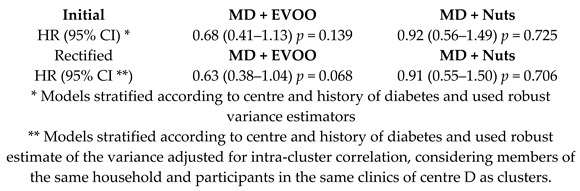	Papadaki et al. *Eur. J. Heart. Fail.* 2017	[20]
				Papadaki et al. *Eur. J. Heart. Fail.* 2019	[21]
Incidence of atrial fibrillation	6705	4.7	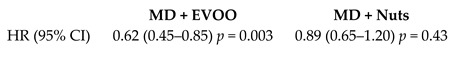	Martínez-González et al. *Circulation* 2014	[22]
**Cardiovascular Risk Factors**					
Long-term consumption of a MD could decrease the atherogenicity of LDL particles	210	1.0	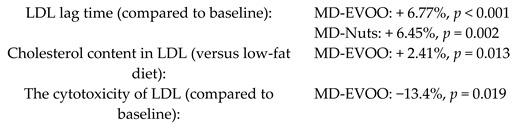	Hernaez et al. *Mol. Nutr. Food Res.* 2017	[23]
Improvement of BP induced by a MD would be mediated by the modulation of NO bioavailability/ET-1 levels	90 Non-smoking women with moderate hypertension	1.0	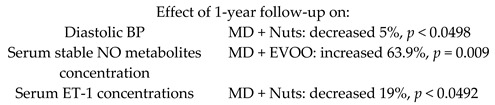	Storniolo et al. *Eur. J. Nutr.* 2017	[24]
Effects of high polyphenol consumption on BP and its relation about production of plasma NO	200	1.0	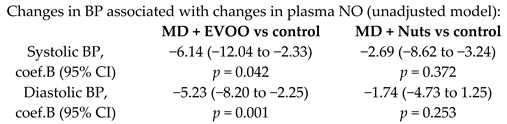	Medina-Remón et al. *Nutr. Metab. Cardiovasc Dis.* 2015	[25]
Effects of MD on inflammatory biomarkers related to atherosclerosis and plaque vulnerability	164	1.0	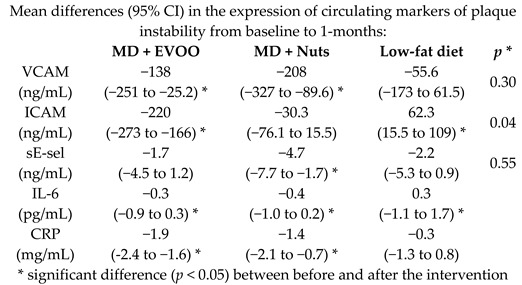	Casas et al. *PLoS ONE* 2014	[26]
MD effect on 24-h ambulatory BP, blood glucose, and lipids	235	1.0	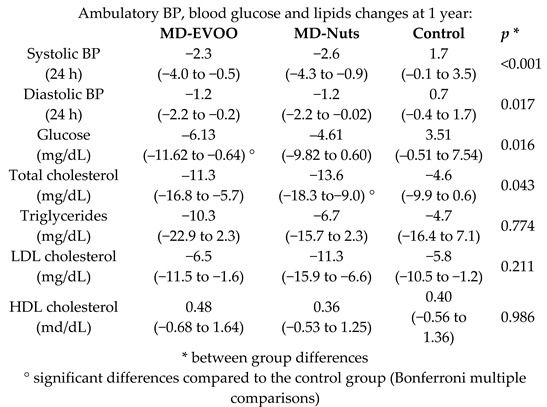	Doménech et al. *Hypertension* 2014	[27]
Effect of the MD on heart failure biomarkers	930	1.0	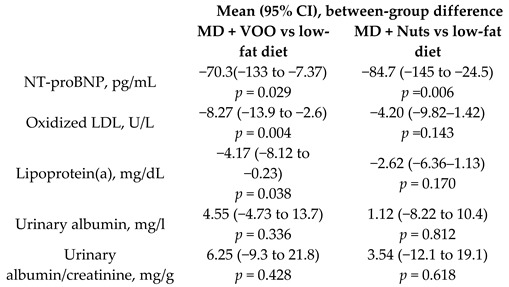	Fitó et al. *Eur. J. Heart Fail.* 2014	[28]
Incidence of Peripheral Artery Disease (PAD)	7435	4.8	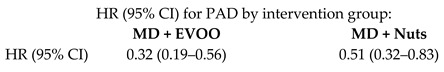	Ruiz-Canela et al. *JAMA* 2014	[29]
Effects of MD on BP	7158	3.8	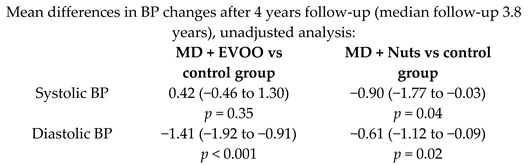	Toledo et al. *BMC medicine* 2013	[30]
Effects of MD on progression of subclinical carotid atherosclerosis	187	1.0	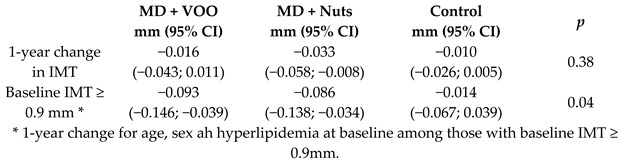	Murie-Fernández et al. *Atherosclerosis* 2011	[31]
The short-term effects of MD versus those of a low-fat diet on intermediate markers of CV risk.	772	0.25	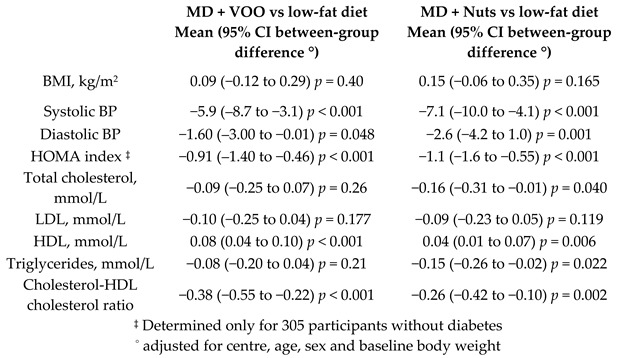	Estruch et al. *Ann. Int. Med.* 2006	[32]

BMI: Body Mass Index; BP: Blood Pressure (mmhg); CV: Cardiovascular; MD: Mediterranean Diet; ET-1: Endothelin 1; EVOO: Extra Virgin Olive Oil; HDL: High-Density Lipoprotein; HOMA: Homeostatic Model Assessment; ICAM: Soluble İntercellular Adhesion Molecule; IL-6: İnterleukin 6; IMT: Intima-Media Thickness; LDL: Low-Density Lipoprotein; MCP-1: Monocyte Chemotactic Protein 1; NO: Nitric Oxide (Um); NT-proBNP: N-terminal pro-brain natriuretic peptide; Se-Sel: Soluble E Selectin; TNF- Α: Tumor Necrosis Factor Alpha; VCAM: Vascular Cell Adhesion Molecule; VOO: Virgin Olive Oil.

**Table 2:** Characteristics of the RCTs conducted within the frame of the PREDIMED study, investigating the role of Mediterranean Diet (MD) on: diabetes mellitus (DM), metabolic syndrome (MetS) and obesity.

Aim of the Study	Number of Subjects	Follow-Up Median (Years)	Main Results of the Study	1st Author, Journal, Year	Ref.
**Diabetes Mellitus**					
Effects of MD versus a low-fat diet on the need for glucose-lowering medications	3230 T2DM	3.2	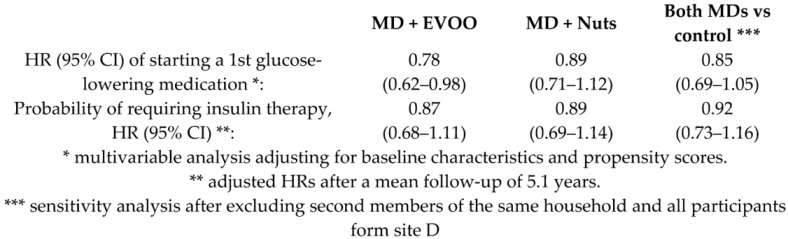	Basterra-Gortari et al. *Diab. Care.* 2019	[33]
Long-term effect of a MD on microvascular diabetes complications	3614 T2DM	6.0	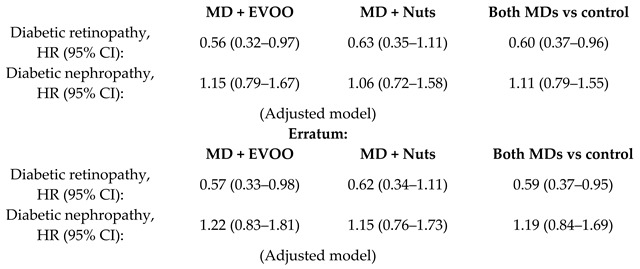	Díaz-López et al. *Diab. Care.* 2015	[34]
				Díaz-López et al. *Diab. Care.* 2018	[35]
Incidence of diabetes	3541	4.1	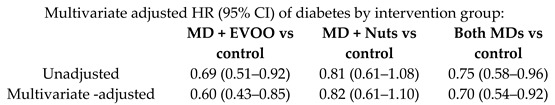	Salas-Salvadó et al. *Ann Int Med.* 014	[36]
**Metabolic Syndrome**					
Plasmatic antioxidant capabilities in Metabolic Syndrome (MetS) patients	75	5.0	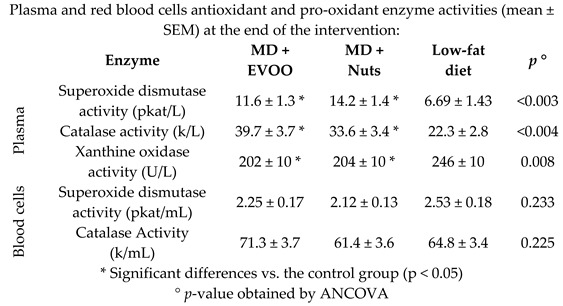	Sureda et al. *Mol. Nutr. Food Res.* 2016	[37]
Long-term effects of MD on MetS	5801	4.8	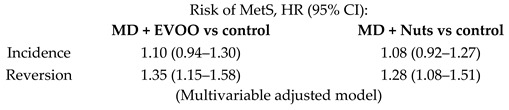	Babio et al. *Cmaj* 2014	[38]
MD effects on MetS status	1224	1.0	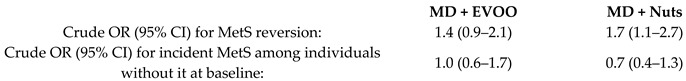	Salas-Salvadó et al. *Arch. Int. Med.* 2008	[39]
**Obesity**					
Effect of a MD on bodyweight and waist circumference	3985	4.8	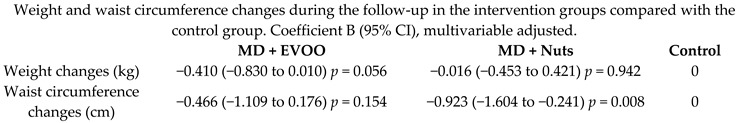	Estruch et al. *The Lancet. Diab. Endocr.* 2019	[40]
Effect of MD on anthropometric variables and body composition parameters	305	1.0	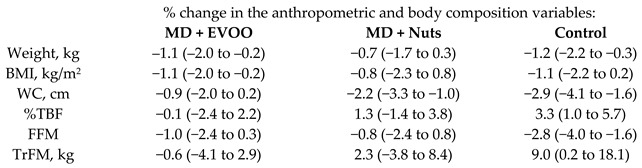	Álvarez-Pérez et al. *J. Am. Coll. Nutr.* 2016	[41]
Effect of MedD on plasma total antioxidant capacity (TAC)	187	3.0	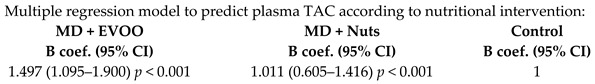	Razquin et al. *Eur. J. Clin. Nutr.* 2009	[42]

BMI: Body Mass Index; CI: Confidence Interval; EVOO: Extra Virgin Olive Oil; FFM: Free Fat Mass; HR: Hazard Ratio; MetS: Metabolic Syndrome; MD: Mediterranean Diet; OR: Odds Ratio; Q: Quartile; TAC: Total Antioxidant Capacity; T2DM: Type 2 Diabetes Mellitus ; TFM: Total Fat Mass; TrFM: Truncal Fat Mass; WC: Waist Circumference; %TBF: percentage of Total Body Fat.

**Table 3:** Characteristics of the RCTs conducted within the frame of the PREDIMED study, investigating the role of Mediterranean Diet (MD) on neurologic disorders and other various conditions.

Aim of the Study	Number of Subjects	Follow-Up Median (Years)	Main Results of the Study	1st Author, Journal, Year	Ref.
**Neurologic Disorders**					
Effect of MD on cognition	522	6.5	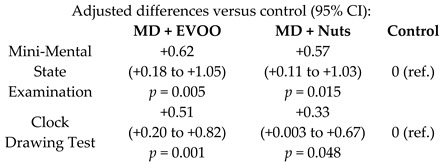	Martínez-Lapiscina et al. *J. Neurol. Neurosurg Psychiatry* 2013	[43]
Effect of MD on Mild Cognitive Impairment (MCI)	268	6.5	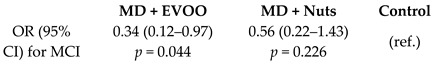	Martínez-Lapiscina et al. *J. Nutr. Health Aging* 2013	[44]
Effects of MD on depression risk	3923	5.4	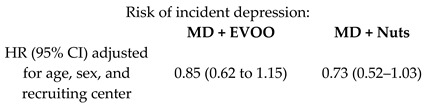	Sánchez-Villegas et al. *BMC medicine* 2013	[45]
Effect of MD on plasma Brain-Derived Neurotrophic Factor (BDNF) levels	243	3	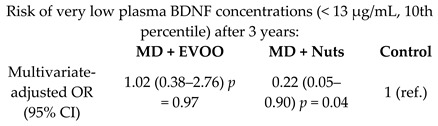	Sánchez-Villegas et al. *Nutr. Neurosci.* 2011	[46]
**Other Conditions**					
MD effect on liver steatosis	100	3.0	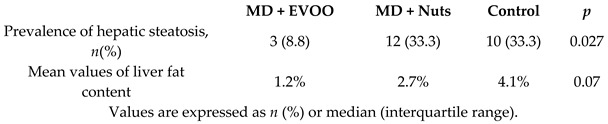	Pintó et al. *J. Nutr.* 2019	[47]
MD effects on the Fatty Liver Index (FLI)	276	6.0	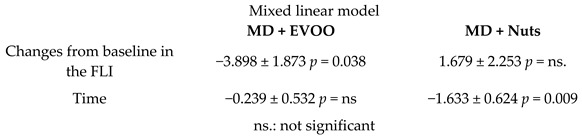	Cueto-Galán et al. *Med. Clin.* 2017	[48]
Incidence of cataract surgery	5802	5.9	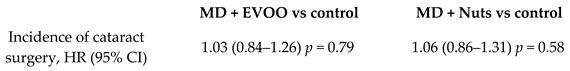	García-Layana et al. *Nutrients* 2017	[49]
Effect of MD on HDL properties	296	1.0	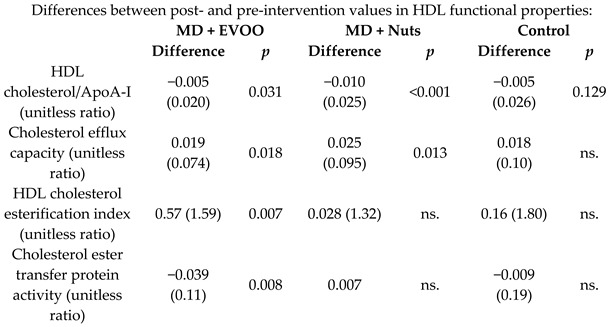 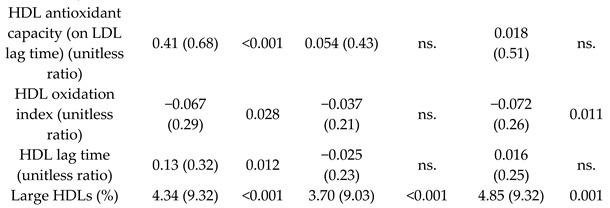	Hernáez et al. *Circulation* 20	[50]
Effect of the MD on inflammatory markers related to atherogenesis	160	3.0 5.0	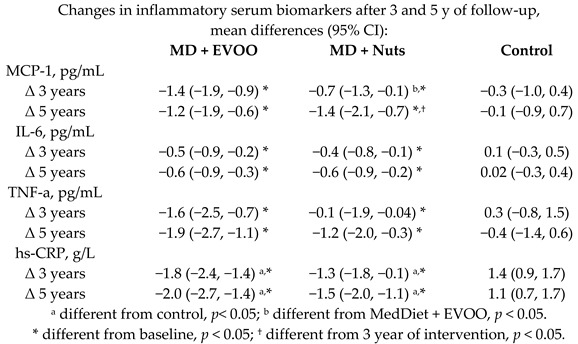	Casas et al. *J. Nutr.* 2016	[51]
Effect of MD on telomere lenght	520	5.0	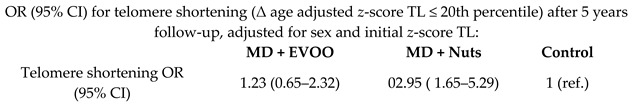	García-Calzón et al. *Clin. Nutr.* 2016	[52]
Breast cancer incidence	4282	4.8	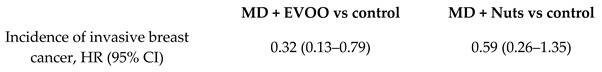	Toledo et al. *JAMA int. Med.*, 2015	[53]
MD effect on lipoprotein subfractions	169	1.0	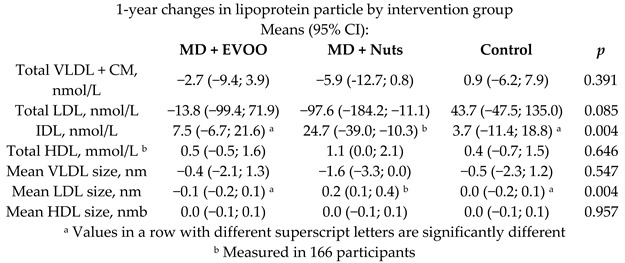	Damasceno et al. *Atherosclerosis* 2013	[54]
Effect of MD on plasma Non-Enzymatic Antioxidant Capacity (NEAC)	564	1.0	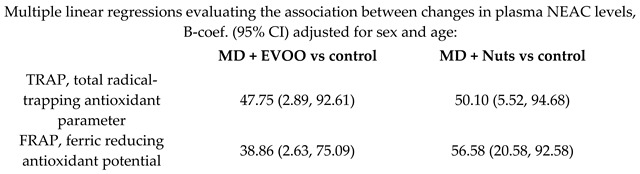	Zamora-Ros et al. *Nutr. Metab. Cardiovasc Dis.* 2013	[55]
Effect of the MD on systemic oxidative biomarkers in MetS individuals	110 female participants with the diagnosis of MetS	1.0	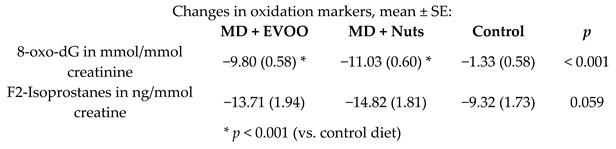	Mitjavila et al. *Clin. Nutr.* 2013	[56]
Effects of MD on apolipoproteins B, A-I, and their ratio	551	0.25	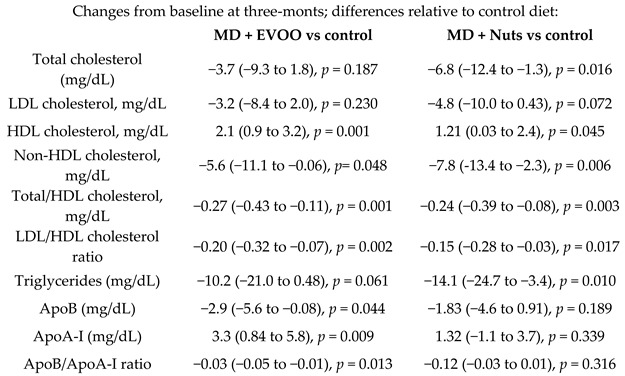	Solá et al. *Atherosclerosis* 2011	[57]
Effects of MD on VLDL concentration	50	0.25	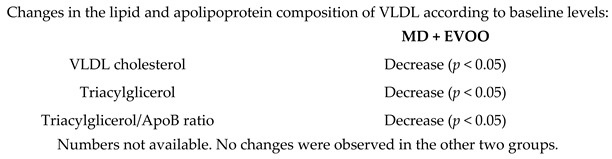	Perona et al. *J. Nutr. Biochem.* 2010	[58]
Phytosterol intake from natural foods association with a cholesterol- lowering effect of MD	106	1.0	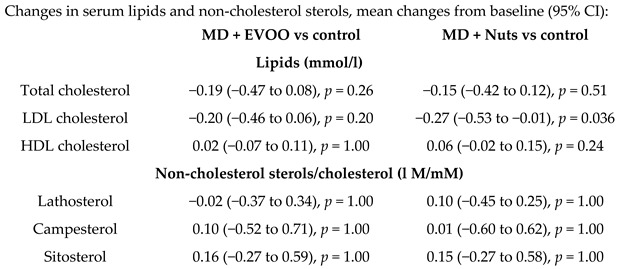	Escurriol et al. *Europ. J. Nutr.* 2009	[59]
Effects of MD on in vivo lipoprotein oxidation	372	0.25	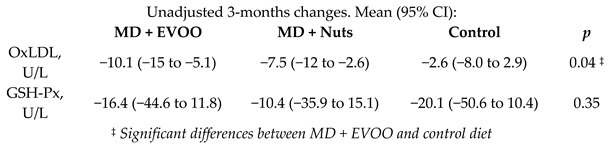	Fitó et al. *Arch. Int. Med.* 2007	[60]

ApoA: Apolipoprotein A; ApoB: Apolipoprotein B; EVOO: Extra Virgin Olive Oil; FLI: Frally Liver Index; GSH-Px: Glutathione peroxidase; HDL: High-Density Lipoprotein; HR: Hazard Ratio; hs-CRP: high sensitivity C-Reactive Protein IDL: Intermediate-Density Lipoprotein; IL-6: Interleukin 6; LDL: Low-Density Lipoprotein; MCP-1: Monocyte Chemotactic Protein 1; MD: Mediterranean Diet; NEAC: Non-Enzymatic Antioxidant Capacity; OR: Odds Ratio; ns: not significant; OxLDL: Oxidized Low-Density Lipoprotein; TNF- Α: Tumor Necrosis Factor Alpha; VLDL: Very-Low-Density Lipoprotein.

**Table 4:** Percentage reduction from the baseline of different continuous variables assessed by the randomized clinical trials in the scope of the PREDIMED study.

		MD + EVOO					MD + Nuts					
Continuous Variable	Time (yr)	N	Mean Value at Baseline	Mean Change	% Change from Baseline	*p*-Value *	N	Mean Value at Baseline	Mean Change	% Change from Baseline	*p*-Value *	Ref.
Sistolic BP (24 h)	1.0	78	127.3	−3.14	−2.5%	-	82	125.3	−2.35	−1.9%	-	[27]
Diastolic BP (24 h)	1.0	78	71.8	−1.68	−2.3%	-	82	71.2	−1.00	−1.4%	-	[27]
BMI, kg/m^2^	0.25	257	29.7	−0.12	−0.4%	-	257	29.4	−0.09	−0.3%	-	[32]
Weight, kg	1.0	112	77.9	−1.0	−1.3%	0.008	102	80.3	−0.5	−0.6%	0.197	[41]
BMI, kg/m^2^	1.0	112	30.7	−0.5	−1.6%	0.012	102	31.2	−0.5	−1.6%	0.314	[41]
WC, cm	1.0	112	100.5	−1.1	−1.0%	0.046	102	102.6	−2.3	−2.2%	<0.001	[41]
Urinary albumin, mg/L	1.0	310	5.0	0.55	11.0%	-	310	5.1	−2.85	−55.9%	-	[28]
Urinary albumin/creatinine, mg/g	1.0	310	7.09	1.13	15.9%	-	310	7.21	−1.62	−22.5%	-	[28]
Intima-media thickness, mm	1.0	66	0.825	−0.016	−1.9%	-	59	0.854	−0.033	−3.8%	-	[31]
Total cholesterol, mg/dL	0.25	181	219.7	−3.7	−1.7%	ns.	193	216.7	−6.8	−3.1%	<0.05	[57]
Oxidized LDL, U/L	1.0	310	74.3	−9.75	−13.1%	-	310	71.1	−5.68	−8.0%	-	[28]
Ox-LDL, U/L	0.25	123	77.9	−10.1	−13.0%	-	128	74.4	−7.5	−10.1%	-	[60]
LDL cholesterol, mg/dL	0.25	181	146.2	−4.3	−2.9%	<0.05	193	141.6	−5.9	−4.2	<0.05	[57]
HDL cholesterol, mg/dL	0.25	181	51.9	1.8	+3.5%	<0.05	193	53.9	0.95	1.8%	<0.05	[57]
Non-HDL cholesterol, mg/dL	0.25	181	174.2	−5.4	−3.1%	<0.05	193	169.6	−7.6	−4.5%	<0.05	[57]
Total/HDL cholesterol, mg/dL	0.25	181	5.0	−0.24	−4.8%	<0.05	193	4.8	−0.20	−4.2%	<0.05	[57]
LDL/HDL cholesterol ratio	0.25	181	3.4	−0.20	−5.9%	<0.05	193	3.1	−0.15	−4.8%	<0.05	[57]
Triglycerides, mg/dL	0.25	181	139.9	−4.8	−3.4%	ns.	193	138.2	−8.62	−6.2%	<0.05	[57]
ApoB, mg/dL	0.25	181	102	−2.8	−4.4%	<0.05	193	101	−1.7	−1.4%	ns.	[57]
ApoA-I, mg/dL	0.25	181	135	2.5	+3.2%	<0.05	193	134	0.16	1.4%	ns.	[57]
ApoB/ApoA-I ratio	0.25	181	0.78	−0.03	−6.2%	<0.05	193	0.78	−0.009	−1.2%	ns.	[57]
Lipoprotein(a), mg/dL	1.0	310	24.8	0.68	2.7%	-	310	24.4	2.23	9.1%	-	[28]
NT-proBNP, pg/mL	1.0	310	572	−27.7	−4.8%	-	310	562	−42.0	−7.4%	-	[28]
GSH-Px, U/L	0.25	123	626	−16.4	−2.6%	-	128	613	−10.4	-1.7%	-	[60]
sVCAM-1, ng/mL	1.0	55	872	−138	−15.8%	0.02	55	935	−208	−22.2%	0.001	[26]
sICAM-1, ng/mL	1.0	55	437	−220	−50.3%	<0.001	55	394	−30.3	−7.7%	0.20	[26]
sE-SEL, ng/mL	1.0	55	28.6	−1.7	−5.9%	0.26	55	33.0	−4.7	−14,2%	0.003	[26]
MCP-1, pg/mL vs. baseline	3.0.	55	4.3	−1.4	−32.6%	<0.05	55	4.6	−0.7	−15.2%	<0.05	[51]
	5.0.			−1.2	−28.0%	<0.05			−1.4	−30.4%	<0.05	
IL-6, pg/mL vs. baseline	3.0	55	1.3	−0.5	−38.4%	<0.05	55	1.4	−0.4	−28.6%	<0.05	[51]
	5.0			−0.5	−46.2%	<0.05			−0.6	−42.9%	<0.05	
TNF-α, pg/mL vs. baseline	3.0	55	3.6	1.6	−44.4%	<0.05	55	3.6	−1.0	−27.8%	<0.05	[51]
	5.0			−1.9	−52.8%	<0.05			−1.2	−33.3%	<0.05	
Hs-CRP, g/L vs. baseline	3.0	55	3.7	−1.8	−48.6%	<0.05	55	3.5	−1.3	−37.1%	<0.05	[51]
	5.0			−2.0	−54.0%	<0.05			−1.5	−42.9%	<0.05	
8-oxo-dG in mmol/mmol creatinine	1.0	38	20.24	−9.80	−48.4%	<0.001	35	19.98	−11.03	−55.2%	<0.001	[56]
F2-Isoprostanes in ng/mmol creatine	1.0	38	76.15	−13.71	−18.0%	-	35	97.40	−14.82	−15.2%	-	[56]

* where not specified, *p*-value is not available due to the computation of the % reduction from baseline of the variables from the available data. .ns.: not statistically significant. BMI: Body Mass Index; BP: Blood Pressure; GSH-px: glutathione peroxidase; HDL: High Density Lipoprotein; hs-CRP: high sentitivity C-Reactive Protein; IL-6: Interleukin 6; LDL: Low Density Lipoprotein; MCP-1: Monocyte Chemotactic Protein 1; NT-proBNP: N-Terminal-pro-Brain Natriuretic Peptide; sE-SEL: soluble E Seclectin; sICAM: soluble Intercellular Adhesion Molecule; sVCAM: soluble Vascular Cell Adhesion Molecule; TNF-α: Tumor Necrosis Factor α; WC: Waist Circumference.
